# In Memoriam—Hays White Moore, M. D.

**Published:** 1886-10

**Authors:** 


### IN MEMORIAM.

### HAYS WHITE MOORE, M. D.

HAYS WHITE MOORE, M. D., son of David B. Moore and Mary White Moore, was born at New Orleans, Louisiana, March 5, 1856, and died at his residence, Fort Worth, Texas, on Sunday, September 26, 1886, of peritonitis, resultant from ulceration of the gall-bladder.

Shortly after the close of the late civil strife (in 1866), when HAYS was but ten years of age, his father and mother died, and he was then placed under the guardianship of his paternal uncle, by whom he was reared.

His early life was spent in New Orleans, where he received the greater part of his academic education, the remainder having been obtained at Emory & Henry College, Virginia.

At the close of his academic course, he visited relations in Tennessee and Kentucky; and while at Pembroke, in the latter State, he made the acquaintance of Dr. Joseph Potts Thomas, a surgeon of wide reputation.

Dr. Thomas was impressed with young MOORE's perception and seeing elements that he thought would develop an able surgeon, he induced him to study medicine. Under the preceptorship of this noted Kentucky surgeon, MOORE progresed rapidly, imbibing many of the traits which lent lustre to the record of his instructor.

Ere long, he entered the Louisville Medical College, and after a due course of study was graduated M. D. therefrom Febuary 27, 1878. During his collegiate term, he received special instruction in gynæcology from Dr. Wm. H. Wathen, and clinical instruction at the various hospitals, receiving also a diploma from the Louisville City Hospital.

Now arrived at manhood, and provided with a physician's armamentarium, DR. MOORE began the practice of his profession in Saddlersville, Tennessee, whence, on account of a limited field of labor, he removed to Fort Worth, Texas, in 1880, where he remained to the time of his death.

In February, 1882, he was appointed city physician of Fort Worth, which position he held until April, 1883, declining re-election, that he might devote more time to private practice. He served as physician and surgeon to the Tarrant county jail from January, 1882, until his death. He was also surgeon to the fire department; local surgeon to the Gulf, Colorado and Santa Fe Railroad; State examiner for the Knights of Honor; county examiner for the order of Chosen Friends, and for several life insurance companies; was a member of the Texas State Medical Association, and chairman of its Section on Surgery (Belton meeting), also a member of its Judicial Council; president of the Fort Worth and Tarrant County Medical and Surgical Association; chairman of the Democratic Executive Committee of Tarrant county, etc.

He contributed to medical literature a paper on "Dementia and Hemiplegia, Resulting from Cerebral Compression, Relieved by Trephining" (read before the Texas State Medical Association, eleventh annual meeting); "Poisoning by Coal

Gas of Two Comanche Chiefs" (DANIEL'S TEXAS MEDICAL JOURNAL, February, 1886), and several other papers.

Prior to his death, he was engaged in the preparation of a "Report of a Successful Laparotomy upon a Female Child, Aged Nine Years." In his earliest experience he exhibited an aptitude for surgery which, had he been permitted the natural allotment of years, would have raised him to the front rank among famous surgeons. Young though he was, ability had already brought to him a recognition for which many strive a lifetime without attaining. The operations he performed were many and various, and among them some of the most difficult in the art of surgery.

For a year or more prior to his death, DR. MOORE had, at times, suffered from hepatic colic; but not until the spring of 1886 had these attacks assumed a serious character. In the latter part of March last, he experienced a long and severe illness, which very nearly proved fatal. After this, he remained in reasonably good health until the following August, when he was again prostrated. He recovered and enjoyed partial immunity from pain until the final attack.

Early Friday morning (September 24th), he was aroused by a severe pain in the right hypochondriac region. The disease progressed rapidly, and by 3 o'clock, a. m., Sunday, his symptoms became alarming. At 6 o'clock, after a consultation, his physicians, Drs. Beall, Adams and Black, concluded he was beyond physical aid. After this, he aroused to consciousness several times. At 3 p. m., he made an effort to get nearer the window; his eyes brightened; one moment of recognition came, a placid smile spread o'er his face—and then his eyes grew meaningless, and he died.

Attended by two loving sisters, and surrounded by friends to whom he was dear as a brother, this grand, noble, princely spirit, took its flight.

An autopsy was made, in the presence of Drs. Beall, Adams, Black, Slauter, and VanZandt, and three friends. It disclosed an ulceration and perforation of the gall-bladder, through which the bile had escaped into the abdominal cavity, causing the fatal peritonitis. A gall-stone, weighing forty-six grains, was found.

The very same condition was presented at the autopsy of the famous St. Louis Surgeon, Prof. John T. Hodgen.

Of commanding appearance and great physical power, nature had made his body a fitting temple for the strong will and powerful mind that lived therein.

Possessing a keen intuitive knowledge of mankind, he was at home among all classes of men. There were none so lowly that they did not feel the effect of his sympathizing presence; none so high that they did not know they had met an equal.

His friends loved him with a devotion inspired by the knowledge that in the hour of danger, and time of trouble, he would be with them with calm courage and strong decision; ready to do battle for their cause; and his enemies turned in silent fear from one who, in the right, knew not the name of peril.

Kind, hospitable and generous; he was a social companion fitted to brighten the hours of revelry and the place of song; and midst the darker scenes of life his charitable hand and open purse were ever ready to alleviate the pangs of misery and want.

His patients found in him not only a doctor, but a friend; for he went to the sick room not as an hireling, but as one who delighted to be the servant of science in her high mission of conquering pain and tribulation, and restoring to health and happiness the afflicted of his race.

If "true religion is to visit the fatherless and widow in their affliction," and in the great unknown, happiness falls to those who loved their brothermen, he has a glorious future beyond the darkness of the grave.

The silent tongues of many mournful hearts do higher tribute to his memory than any effort of this pen; for years shall fade away, and old men and women, one by one go tottering to the tomb, ere from the minds of this people will pass away the cherished memory of DR. HAYS WHITE MOORE. J.-H.

Fort Worth, Texas, Oct. 12, 1886.

